# Charge Recombination Deceleration by Lateral Transfer of Electrons in Dye-Sensitized NiO Photocathode

**DOI:** 10.1021/jacs.3c00269

**Published:** 2023-05-16

**Authors:** Chen Ye, Haoliang Cheng, Sina Wrede, Stéphane Diring, Haining Tian, Fabrice Odobel, Leif Hammarström

**Affiliations:** † Department of Chemistry-Ångström Laboratories, Uppsala University, Uppsala SE75120, Sweden; ‡ CNRS, CEISAM UMR 6230, Université de Nantes, F-44000 Nantes, France

## Abstract

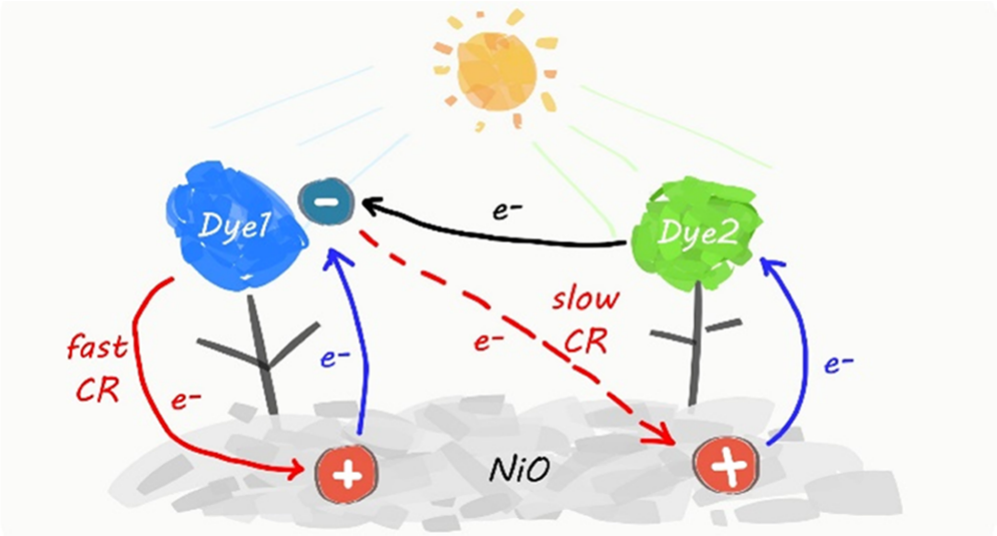

Control of charge separation and recombination is critical for dye-sensitized solar cells and photoelectrochemical cells, and for p-type cells, the latter process limits their photovoltaic performance. We speculated that the lateral electron hopping between dyes on a p-type semiconductor surface can effectively separate electrons and holes in space and retard recombination. Thus, device designs where lateral electron hopping is promoted can lead to enhanced cell performance. Herein, we present an indirect proof by involving a second dye to monitor the effect of electron hopping after hole injection into the semiconductor. In mesoporous NiO films sensitized with peryleneimide (PMI) or naphthalene diimide (NDI) dyes, dye excitation led to ultrafast hole injection into NiO from either excited PMI* (τ < 200 fs) or NDI* (τ = 1.2 ps). In cosensitized films, surface electron transfer from PMI^–^ to NDI was rapid (τ = 24 ps). Interestingly, the subsequent charge recombination (ps−μs) with NiO holes was much slower when NDI^–^ was generated by electron transfer from PMI^–^ than when NDI was excited directly. We therefore indicate that the charge recombination is slowed down after the charge hopping from the original PMI sites to the NDI sites. The experimental results supported our hypothesis and revealed important information on the charge carrier kinetics for the dye-sensitized NiO photoelectrode system.

## Introduction

Substantial research efforts have been devoted to understanding the fundamental behavior of dye-sensitized solar cells (DSSCs).^1^ Upon light absorption, sensitizers form their excited states and subsequently inject free charges into the corresponding semiconductor matrix. By charge transfer from a dye to a redox couple, the remaining interfacial charge can be finally utilized in solar energy conversion.^2^ For p-type NiO DSSCs, charge recombination represents a great problem and still limits the charge collection efficiency.^3−7^ The charge recombination lifetime in many NiO DSSCs occurs predominantly on the picosecond time scale. Pioneering studies on n-type (mainly TiO_2_) DSSCs have demonstrated that the addition of a secondary electron donor to the dye, to shift the hole further from the semiconductor after electron injection, can significantly slow down recombination.^8−11^ The corresponding approach with dye–acceptor dyads on NiO led to as much as *ca*. five orders of magnitude retardation of charge recombination moving from an ∼100 ps to an ∼10 μs time scale;^7,12,13^ related effects have since been reported.^14−16^ The large difference cannot be simply explained in terms of variation in reaction free energy or the distance factors. Furthermore, with dyes and molecular proton reduction catalysts co-adsorbed on NiO, rapid (∼10 ps) electron transfer from the dye to the catalyst was observed after hole injection, which led to charge recombination on the ∼10 μs time scale.^17,18^ This strong retardation is difficult to explain just based on the properties of the dyes and catalysts. D’Amario et al. found that the injected holes in NiO can relax on a 10–100 ns time scale to defect sites and become less active.^19−21^ As the holes delocalize in the NiO, the electrons also migrate onto the dye layer.^19,20,22,23^ We can speculate that charge hopping removes the charge from the original sites and makes the electron–hole pairs uncorrelated. The geminate recombination is then suppressed, and more charge separation states survive until the hole relaxation. We therefore hypothesized that electron transfer between dye molecules in a NiO DSSC has the potential to suppress the charge recombination and prolong the lifetime of the charge separation states, which would explain the previous results. While isoenergetic electron hopping between the same dye molecules in electrochemical experiments was found to be slow (*k*_hop_ ∼ 10^5^ s^–1^)^23^ and may not be able to compete efficiently with charge recombination, electron transfer to secondary acceptors in the above examples has been very fast (∼10 ps) and may quickly decorrelate electrons from injected holes.

To test the hypothesis, we designed a NiO DSSC system with tunable electron transfer among the lateral dyes. We prepared a NiO mesoporous film according to the reported sol–gel doctor blading method. The NiO films are mainly composed of NiO nanoparticles at around 10 nm and are rich in surface states (Figure S1).^24^ The holes migrate within the NiO while the electrons diffuse at the outer surface along the isolated dye sites.^23,25,26^ The above factors lead to the incongruous motion of holes and electrons and separate them in space. Since the holes are moving inside the NiO sphere and hop through the defect sites, we can then mainly focus on the lateral electron transfer between the dye molecules.

**Scheme 1:**
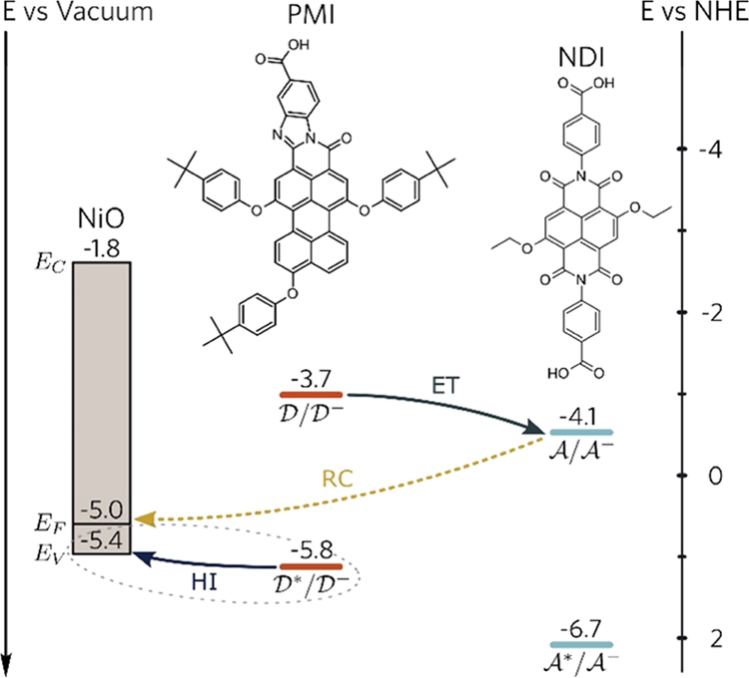
Energy Diagram of NiO, PMI (D), and NDI (A) and the Hypothesized Mechanism of Lateral Electron Transfer (ET: electron transfer; HI: hole injection; RC: recombination)

The lateral electron transfer among the same molecule species in a binary NiO–dye system is difficult to observe directly in optical spectroscopy due to the homogeneity. Indirect methods including spectroelectrochemistry and transient photoinduced anisotropy of absorption can reveal the kinetic information of homogeneous electron hopping.^23,27−32^ Alternatively, we expect to find the correlation between lateral electron transfer and recombination in a direct way by involving a second dye molecule species as an electron acceptor. Once the electrons hop from the donor site to the acceptor site, a new reduced species could be monitored by optical spectroscopy. When we excite the donor molecules D in a ternary system NiO-D–A, the injected holes will be located close to the molecules D positions (Scheme 1). We, therefore, assume that the subsequent lateral electron transfer from D^–^ to acceptor molecules A and holes will slow down the overall recombination since the electron–hole distance is enlarged.

## Results and Discussion

**Figure 1:**
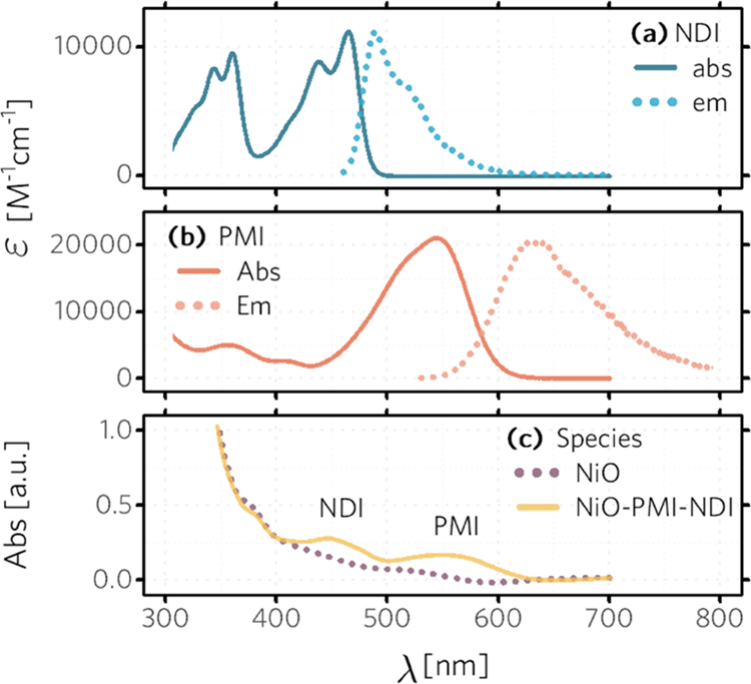
Absorption and emission spectra of (a) NDI in DMF solution (b) PMI in THF solution; (c) absorption spectra of a NiO film and NiO–PMI–NDI ternary film.

Considering the energetic requirement in a proposed model, we involve perylene-1,6,9-tri(4-tert-butylphenoxy)-9-bromo-3,4-(4-carboxylic acid-1,2-benzimidazole) (PMI) as the electron donor and *N*,*N*′-di(4-carboxylic acid)-3,7-ethoxy-1,4,5,8-naphthalenediimide (NDI) as the electron acceptor based on previous results.^12,34,35^ The reduction potential (M^–^/M) for PMI and NDI is −0.98 and −0.56 V vs saturated calomel electrode (SCE), respectively. The electron transfer from PMI^–^ to NDI is then thermodynamically allowed. The *E*_00_ energy of PMI (2.11 eV) is much lower than that of NDI (2.61 eV), with no spectral overlap between the absorption of NDI and the emission of PMI (Figure 1a,b). We can then excite the two dyes PMI and NDI directly at 480–550 and 410–450 nm, respectively. Because of the large excitation energy difference, the energy transfer from PMI* to NDI is excluded. Both dye molecules were substituted with the carboxylic acid group at the terminals, in order to chemically anchor the dye to NiO.^36^ By comparing the absorption spectra of NiO film and NiO film immersed in a dye bath overnight, we ensure the loading dye molecules onto the NiO surface. The mean thickness of the NiO film was measured to be 780 nm (Figure S2). The surface-loaded NDI and PMI on the NiO electrode in Figure 1 were estimated to be 3.7 × 10^19^ and 2.4 × 10^19^ cm^–3^, respectively.

**Figure 2:**
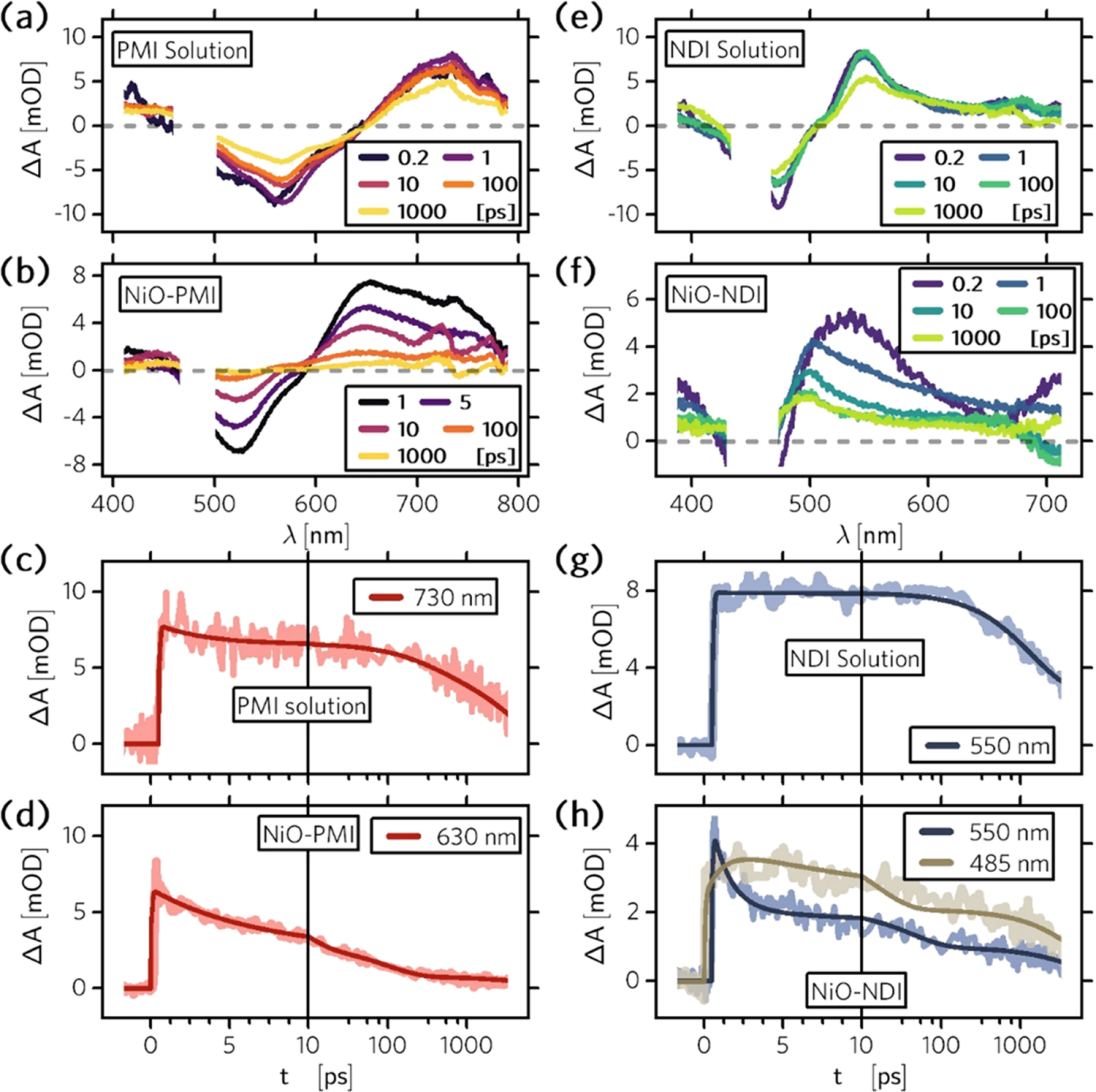
Femtosecond transient absorption spectra (a–d) and kinetic traces (e–h) of dye solutions and binary system films. Transient absorption spectra of (a) PMI solution excited at 470 nm (370 nJ/pulse), (b) NiO–PMI film excited at 470 nm (610 nJ/pulse), (e) NDI solution excited at 440 nm (330 nJ/pulse), and (f) NiO–NDI film excited at 440 nm (580 nJ/pulse); time-resolved transient absorption traces at characteristic wavelengths for (c) PMI solution, (d) NiO–PMI film, (g) NDI solution; and (h) NiO–NDI film.

To investigate the hole injection kinetics, we prepared two kinds of binary system films, NiO–PMI and NiO–NDI, and excited them with ∼200 fs laser pulses at 470 and 440 nm, respectively. Compared with the femtosecond transient absorption spectra of the dye solutions, we confirmed the fast hole injection into NiO and the formation of reduced dye species (Figure 2, Figures S3-S4). The PMI solution shows an induced absorption (IA) from its first singlet excited state at around 730 nm, while the NiO–PMI binary film shows an instant appearance of the IA at around 630 nm. The blue-shifted IA in NiO–PMI film is in accordance with the electronic absorption of the single reduced species PMI^–^ in solution (Figure S5) and with previous work on PMI–NiO.^12,34^ The formation of this signal is mostly within the instrument response, indicating an ultrafast hole injection (*k* > 5.0 × 10^12^ s^–1^, τ < 200 fs) from PMI* to NiO. However, a significant part of the excited state absorption around 700 nm is still present at 1 ps and decays on the time scale of a few picoseconds. The TA dynamics thus shows at least biphasic hole injection, in agreement with previous reports.^12,13,34^

The NDI shows the singlet excited-state absorption at around 550 nm in solution (Figure 2e), in agreement with the literature,^37^ but on NiO–NDI binary films, the initial IA from the *NDI excited state around 550 nm shifts to around 480 nm within a few picoseconds (Figure 2f); this latter signal is characteristic of the NDI^–^ radical.^13,38^ The excited-state decay kinetics is in agreement with the buildup of the NDI^–^ signal. The rate constant for hole injection from NDI* to NiO is then calculated to be 8.4 × 10^11^ s^–1^ (τ = 1.2 ps). The high hole injection rates for both samples ensure the effective charge separation of the NiO–dye system and thus provide us the possibility to investigate the following charge kinetics in our hypothesized model.

The initial part of charge recombination in NiO–PMI, as monitored by the PMI^–^ signal decay (Figure 2d), shows very heterogeneous kinetics with components ranging from tens of ps to tens of ns. This is typical for dye-sensitized NiO.^5,34^ Interestingly, the NDI^–^ signal in NiO–NDI (Figure 2h) is only decaying slightly on the time scale of <1 ns. Charge recombination at longer time scales was therefore monitored by nanosecond transient absorption spectroscopy with excitation pulses of about 10 ns duration (Figure S6). Both the radical anions PMI^–^ and NDI^–^ can survive up to 10^–5^ s before recombination with holes in NiO is complete.

With the kinetic parameters from the binary systems, we further expanded our investigation to the ternary system NiO–PMI–NDI. The generic case of pairwise interactions must be considered, with the presence of multiple chromophores. Excitation energy transfer from PMI* to NDI is excluded, since the NDI has a much higher *E*_00_.^34,35^ We exclude direct photoinduced electron transfer between the dyes in the film system(*PMI–NDI → PMI^+^–NDI^–^), due to the fast hole injection into NiO, the absence of any PMI^+^ features in the TA spectra,^12^ and the clear observation of PMI^–^ to NDI^–^ conversion (see below).

Based on the preconditions proven above, we can finally test our hypothesis by comparing the direct recombination by exciting NDI and the indirect recombination by exciting PMI in the same ternary system NiO–PMI–NDI film. The molecular coordinates are identical since we are comparing the different kinetic processes on the same sample and can ignore molecular migration due to the chemical anchoring effect. When NDI chromophores are excited at 440 nm in the ternary system, we predominantly observed direct hole injection, followed by recombination between NDI^–^ and holes in NiO. The kinetic process of NiO–PMI–NDI^–^ is similar to the case of the binary system NiO–NDI^–^, since NDI^–^ is not reactive to PMI (Figure S7). The absence of the bleach of PMI at 550 nm also indicates that energy transfer from NDI* to PMI is not significant (Figure S8).

**Figure 3:**
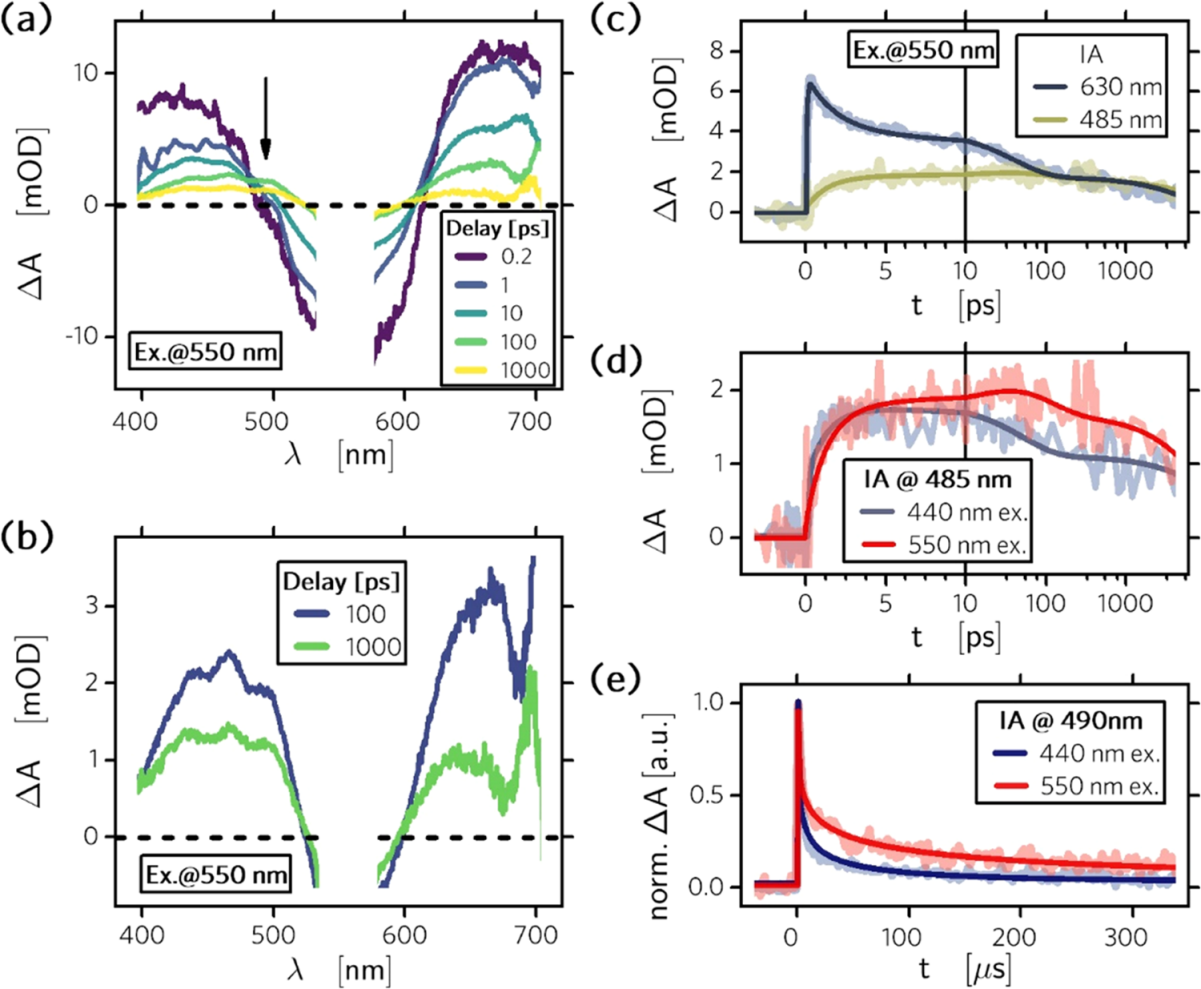
Transient absorption spectra of the ternary system NiO–PMI–NDI film. (a,b) Femtosecond transient absorption spectra of the NiO–PMI–NDI film excited at 550 nm with a pulse energy of 1.33 μJ. IA decay with the overlaid fit of the (c) NiO–PMI–NDI film at 485 and 650 nm excited at 550 nm with 1.33 μJ; (d) NiO–PMI–NDI film at 485 nm excited at 440 nm with 1.17 μJ and 550 nm with 1.33 μJ; and (e) NiO–PMI–NDI film at 490 nm excited at 440 nm with 19.2 mJ and 550 nm with 31.0 mJ.

The excitation of PMI chromophores at 550 nm in the ternary system led to the instantaneous formation of PMI^–^ by hole injection, observed by the IA at around 650 nm (Figure 3a). The IA band of NDI^–^ at around 490 nm was subsequently observed after a few picoseconds (Figure 3a,b), indicating the occurrence of electron transfer from PMI^–^ to NDI on the NiO surface of the ternary system. The NDI^–^ extinction coefficient is only about one-third of that for PMI^–^ at 630 nm;^38^ thus, the electron transfer yield is close to 100% (ca. 7 mOD decrease at 630 nm from 1 to 100 ps and somewhat more than 2 mOD increase at 490 nm). Unlike the self-exchange hopping among the same molecular species, the electron “hopping” to the different molecular species can be identified and monitored directly by the new electronic absorption peak. Comparing the NDI^–^ formation and PMI^–^ decay, we calculated the surface electron transfer rate constant from PMI^–^ to NDI to be 4.2 × 10^10^ s^–1^ (τ = 24 ps, SI I.C, Table S1). The electron transfer reaction thus occurs mainly on the sub-nanosecond time scale. A similarly fast electron transfer between surface-attached molecules has been reported in related systems, with coumarin dyes (C343) and an iron catalyst complex in NiO.^17,18^

The recombination between NDI^–^ and holes in NiO can be traced by recording the decay of NDI^–^. The geminate electron/hole pairs are separated by electron transfer away from the original sites. Most importantly, we observed a slower decay of the NDI^–^ signal at 490 nm after exciting the PMI chromophores than after exciting the NDI chromophores in the same sample (Figure 3d,e). The difference between the direct and indirect recombination is evident already on the picosecond time scale and is very large at the nanosecond to microsecond time scale. The ultrafast decay components have lifetimes of 52 ps (*k* = 1.9 × 10^10^ s^–1^) for direct recombination by exciting NDI and 86 ps (*k* = 1.2 × 10^10^ s^–1^) for indirect recombination by exciting PMI. Considering the disorder relaxation at the longer time scale, we used the Kohlrausch–Williams–Watts (KWW) function to fit the transient absorption decay and obtained average lifetimes of 26.3 μs (*k* = 4.1 × 10^4^ s^–1^, β = 0.31) for direction recombination and 188 μs (*k* = 4.8 × 10^3^ s^–1^, β = 0.28) for indirect recombination (Figures 3e and S9).^39,40^ The indirect recombination of the ternary system is also much slower than the recombination NiO–NDI binary system (Figure S10). We also observed that the recombination is independent of the excitation intensity, as is clear from a comparison of normalized data (Figure S11).

Since we can monitor the recombination from the same electron-containing species (NDI^–^) to the holes in the NiO, the only variable involved here is the initial separation of the electron–hole pair and any further displacement that occurs subsequently. We can conclude that surface electron transfer can delay the overall recombination in the NiO–dye system. This may suggest an important effect of geminate recombination on NiO, which is delayed by the rapid separation of the geminate pair in the NDI–PMI–NiO experiments. This is in contrast to the general belief that non-geminate recombination dominates in NiO–dye systems, which is supported by the large density of states near the NiO conduction band already in the dark and the observation of a strong potential bias dependence of recombination.^6,14,15,19,41^ We note that the potential bias in previous studies rather affected the amount of the reduced dye observed but did not change its lifetime on a time scale up to ∼10 ns.^14,19,41^ It is thus conceivable that the bias in many cases affects the initial charge separation into a distinguishable product rather than the lifetime of subsequent recombination.^6,14,15^ This means that the effect of bias and non-geminate recombination on ps time scales may need reconsideration.

We thus extend our conclusion to the more general self-exchange hopping and predict that surface electron hopping among the dyes on NiO can delay the overall recombination with the holes in the semiconductor electrode, with proper kinetic parameters. With the multiple-step hopping between NDIs, the holes inside NiO will gain time to relax to the less reactive sites, which is more important for the charge separation in the p-DSSC system.^19,21,42^ Indeed, DSSCs with NiO–PMI–NDI photoelectrodes were tested and showed higher absorbed-photon-to-current efficiency (APCE) in the absorption band of PMI than in that of NDI (Figure S12). This can be attributed to the prolonged lifetime of NDI^–^ generated by PMI excitation and lateral electron transfer, which can give a higher yield of dye regeneration by the electrolyte redox couple (I_3_^–^/I^–^).

## Conclusions

In conclusion, we made a hypothesis that electron hopping between the dyes on the NiO surface can delay the recombination in dye-sensitized photoelectrodes. We designed a ternary system NiO–PMI–NDI, where both the dyes can efficiently inject holes into NiO after proper excitation. The influence of surface electron transfer can be then represented by PMI^–^ to NDI, with the electronic absorption band of NDI^–^ to be monitored. The direct recombination of NDI^–^ → holes is much faster than the indirect recombination of PMI^–^ → NDI^–^ → holes. The dye–dye electron transfer can reduce the electron/hole correlations in space and give time for the holes to be relaxed to less reactive sites of NiO. These effects will compress the overall recombination. We therefore verified the hypothesis in the work. The effect of dye surface concentration and spatial arrangement will be important considerations in future studies aiming to expand on this topic. Our results suggest that geminate recombination is important on the ps time scale and is a loss factor in most NiO-based DSSCs. This is in contrast to the generally proposed dominance of non-geminate recombination, as discussed before. The conclusions provide us with a fundamental insight into the charge carrier kinetics of p-DSSCs and help us understand better the current challenges for charge separation and utilization with dye–semiconductor photoelectrodes.

## Supporting Information Available

Experimental details, fluorescence quenching, additional ns-TAS and fs-TAS data, fitting procedures, and IPCE test (PDF)
