# Supplementary material for: Charge Recombination Deceleration by Lateral Transfer of Electrons in Dye-Sensitized NiO Photocathode
Source: J Am Chem Soc. 2023 May 16;145(20):11067–73. doi: 10.1021/jacs.3c00269 (PMC10214442; doi:10.1021/jacs.3c00269)
Supplement: Supplementary file 1 — ja3c00269_si_001.pdf [file ja3c00269_si_001.pdf]

Supporting Information
Charge Recombination Deceleration by Lateral Transfer of Electrons in Dye Sensitized NiO Photocathode

Chen Ye,[1] Haoliang Cheng,[1] Sina Wrede,[1] Stéphane Diring,[2]
Haining Tian,[1,*] Fabrice Odobel,[2,†] and Leif Hammarström[1,‡]

[1]*Department of Chemistry-Ångström Laboratories,*
Uppsala University, Uppsala SE75120, Sweden

[2]*CNRS, CEISAM UMR 6230, Université de Nantes, F-44000 Nantes, France.*

CONTENTS

I. Experiment Section 2
A. NiO Film Preparation 2
B. NiO-dye Film Preparation 2
C. Steady State Spectroscopy 3
D. Nanosecond Transient Absorption Spectroscopy 3
E. femtosecond Transient Absorption Spectroscopy 3

II. Mathematical Methods 4
A. Fitting details in Nanosecond Transient Absorption 4
B. Fitting details in Femtosecond Transient Absorption 4
C. Hole Injection Electron Transfer and Recombination Kinetics 5
D. Programs for Data Analysis 5

III. Supporting Figures and Tables 6

References 13

I. EXPERIMENT SECTION

A. NiO Film Preparation

Mesoporous NiO films were prepared by a doctor-blading method on the substrate of fluorine-doped tin oxide (FTO) glass. The precursor sol-gel was prepared by the literature methods.[1] The dye files were then annealed at 450 °C for 30 min to form the final NiO mesoporous film. The PMI and NDI dyes were available from previous studies.[2, 3]

B. NiO-dye Film Preparation

NiO-PMI and NiO-NDI films were prepared by immersing the mesoporous NiO film in to 17 μM THF solution of PMI and 25 μM DMF solution of NDI overnight, respectively.

* haining.tian@kemi.uu.se
† Fabrice.Odobel@univnantes.fr
‡ leif.hammarstrom@kemi.uu.se

The film samples were then rinsed by ethanol and dried with argon.

NiO-PMI-NDI films were prepared by two-step loading. NDI was first loaded by same way of NiO-NDI. PMI was loaded afterwards, due to the extremely poor solubility of NDI in THF. All the loading process was operated at dark environment. All the prepared films were kept at dark condition.

C. Steady State Spectroscopy

The absorption measurements were carried out at a UV-Vis spectrometer (Cary 5000, *Agilent Technologies*). The emission measurements were carried out at a photoluminescence spectrometer (Fluorolog-3 fluorimeter, *Horiba Scientific*).

D. Nanosecond Transient Absorption Spectroscopy

The ns-transient absorption measurements were carried out at a commercial transient absorption spectrometer (LP920, *Edinburgh Instruments*). The pump source is a Q-switched Nd:YAG laser (Model NT342B, *EKSPLA*; repetition rate: 10 Hz, FWHM = 8 ns) with fundamental emission at 1064 nm. The pump pulse was induced into pump an optical parametric oscillator (OPO) to generate the desired wavelength (440 or 550 nm). Spectral data was recorded by a CCD camera (iStar, *Andor*), and the kinetic data was recorded by a photomultiplier tube (R928, *Hamamatsu*). The acquisition rate for both modes were 1 Hz. The probe light was porived by a Xe arc lamp at pulsed mode. The pump-probe geomtry was set at the standard configuration for transient absorption. The sample film was set at 45° to the probe path.

E. femtosecond Transient Absorption Spectroscopy

The fs-transient absorption measurements were carried out at a combined system of commercial components. The laser source was provided by an amplified Ti:Sapphire laser (Libra, *Coherent*) The fundamental output has a repetition rate of 3 kHz, with $\lambda = 800$ nm. The fundamental beam was splitted into two beams. One beam was transformed into the probe light through a CaF_2 crystal with continuous moving. The other beam was induced

to into an optical parametric amplifiers (TOPAS-prime, *Light Conversion*) to generate the desire pump wavelength (440 nm, 470 nm, and 550 nm). The samples were located inside the UV-vis-NIR sample chambers (TAS, *Ultrafast Systems*) at the reverse mode under magic angle polarization. The time control was achieved by a mechanical optical delay stage, with a scan range -5 ps $\sim$ 8 ns. The instrument response is at ca. 150 fs.

II. MATHEMATICAL METHODS

A. Fitting details in Nanosecond Transient Absorption

The nanosecond transient absorption decay of solution samples and highly-odered film samples can be well fitted in an exponential decay model:

$$\begin{cases} A(t) & = \sum A_i \exp\left(-\frac{t}{\tau_i}\right) \\ \langle\tau\rangle & = \frac{\sum A_i \tau_i^2}{\sum A_i \tau_i} \end{cases} \tag{1}$$

The nanosecond transient absorption decay of film samples with disordered relaxation can be fitted in the KWW function[4]:

$$\begin{cases} A(t) & = A \exp\left[-\left(\frac{t}{\tau}\right)^{\beta}\right] \\ \langle\tau\rangle & = \frac{\tau}{\beta}\Gamma\left(\frac{1}{\beta}\right) \end{cases} \tag{2}$$

The stretched exponential function with the stretch parameters β can be considered as a linear superposition of simple exponential decays. The lifetimes follows a nontrivial and continuous distribution[5]:

$$G(t) = -\frac{1}{\pi}\sum_{k=0}^{\infty}\frac{(-1)^k}{k!}\sin(\pi\beta k)\Gamma(\beta k+1)\left(\frac{t}{\tau}\right)^{\beta k} \tag{3}$$

B. Fitting details in Femtosecond Transient Absorption

Chirp correction was performed by fitting the coherence spiking at the initial stage close to time zero[6]. The decay kinetics follows the convolution result of an exponential decay model and a Gaussian type instrument response function (IRF):

$$F(t) = \left(\sum A_i e^{-\frac{t-t_0}{\tau_i}}\right) \otimes e^{-\left(\frac{t-t_0}{\beta}\right)^2} \tag{4}$$

where the full width at half maximum (FWHM) of IRF is : $\beta \ln 4$.

Analysis of multi-component kinetics can be complicated, especially when the decay components possess a wide range distribution of lifetimes. The average lifetime is calculated by a modified weighted average lifetime methods to avoid giving bias to the slow components[7]:

$$\langle \log(\tau_{avg}) \rangle = \frac{\sum A_i \log(\tau_i)}{\sum A_i} \tag{5}$$

The modified equation for average lifetime gives a fair consideration on lifetimes at different magnitudes. It also satisfies the way to calculate the rate constant of the parallel process, since $(\sum A_i \tau_i)^{-1}$ is not identical to $\sum A_i k_i$:

$$\langle \log\, \tau \rangle^{-1} = \langle k \rangle \tag{6}$$

C. Hole Injection Electron Transfer and Recombination Kinetics

The hole injection kinetics from the first singlet excited state of dye $\mathcal{M}$ of into NiO follows:

$$\frac{\mathrm{d}}{\mathrm{d}t}\begin{bmatrix} \mathcal{M}^* \\ \mathcal{M}^- \end{bmatrix} = \begin{bmatrix} -(\sum k_s + k_{HI}) & 0 \\ k_{HI} & -k_{CR\mathcal{M}} \end{bmatrix} \begin{bmatrix} \mathcal{M}^* \\ \mathcal{M}^- \end{bmatrix} \tag{7}$$

The kinetics of the ternary system involves the lateral electron transfer from PMI$^-$ ($\mathcal{D}^-$) to NDI ($\mathcal{A}$), and thus follows :

$$\frac{\mathrm{d}}{\mathrm{d}t}\begin{bmatrix} \mathcal{D}^* \\ \mathcal{D}^- \\ \mathcal{A}^- \end{bmatrix} = \begin{bmatrix} -(\sum k_s + k_{HI}) & 0 & 0 \\ k_{HI} & -(k_{CR\mathcal{D}} + k_{ET}) & 0 \\ 0 & k_{ET} & -k_{CR\mathcal{A}} \end{bmatrix} \begin{bmatrix} \mathcal{D}^* \\ \mathcal{D}^- \\ \mathcal{A}^- \end{bmatrix} \tag{8}$$

The build up process of NDI$^-$ ($\mathcal{A}^-$) reflects the lateral electron transfer from PMI$^-$ ($\mathcal{D}^-$) to NDI ($\mathcal{A}$). The lateral electron transfer rate constant (k_{ET}) can be therefore calculated by fitting the transient absorption data with eq.8.

D. Programs for Data Analysis

The data analysis was mainly done by own script written in *R* (Version 4.0.3) with packages "*Tidyverse*"," *deSolve*" and "*minpack.lm*". All the kinetic traces were fitted by least squares methods with Levenberg-Marquardt algorithm.

III. SUPPORTING FIGURES AND TABLES

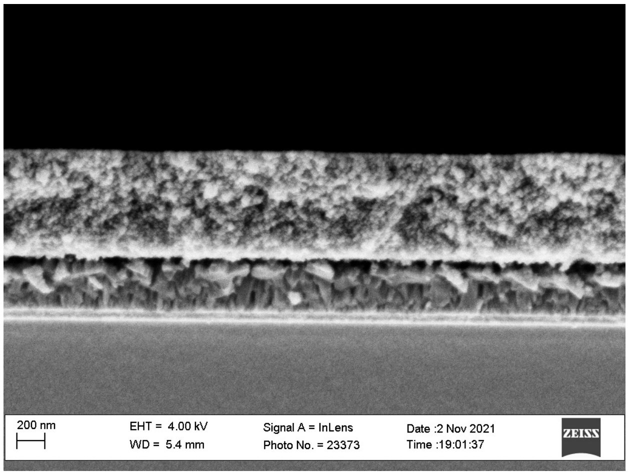

FIG. S1. SEM image of the NiO electrode.

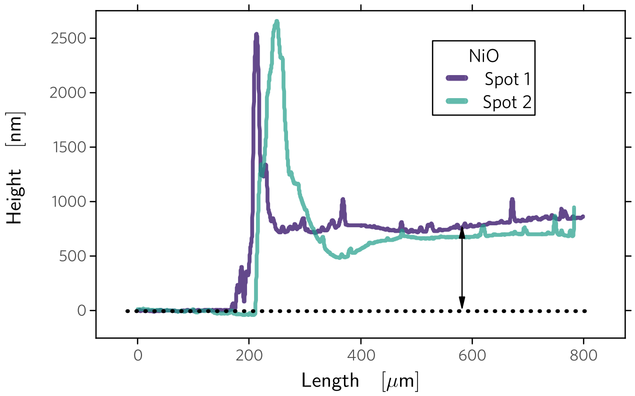

FIG. S2. The profilometer result of NiO.)

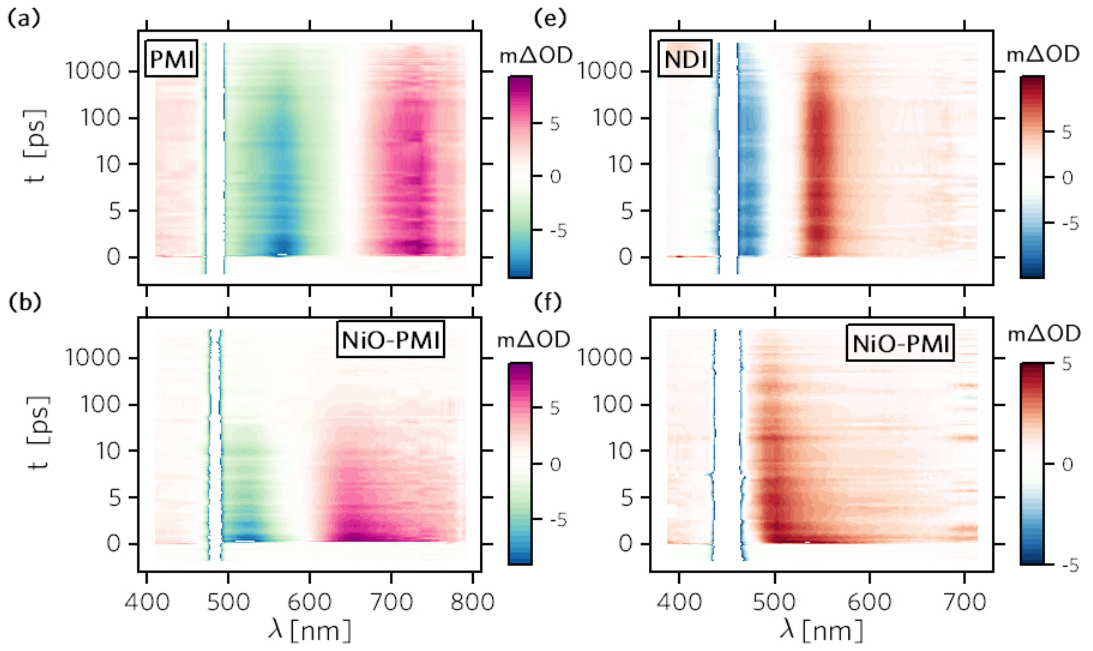

FIG. S3. Femtosecond transient absorption map of PMI solution and NiO-PMI film excited at 440 nm and NDI solution and NiO-NDI film excited at 470 nm.

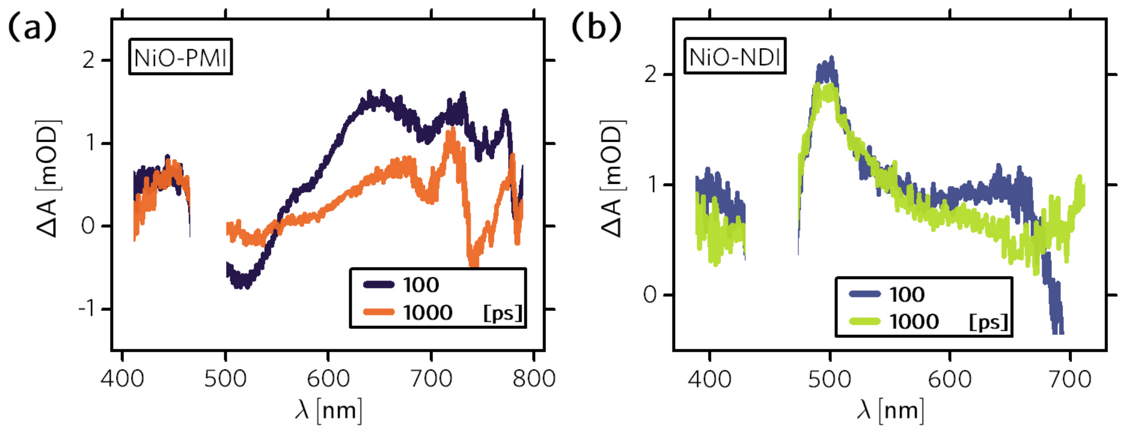

FIG. S4. Femtosecond transient absorption spectra of NiO-PMI film excited at 440 nm and NiO-NDI film excited at 470 nm at longer probe delay time (100 ps, 1 ns).

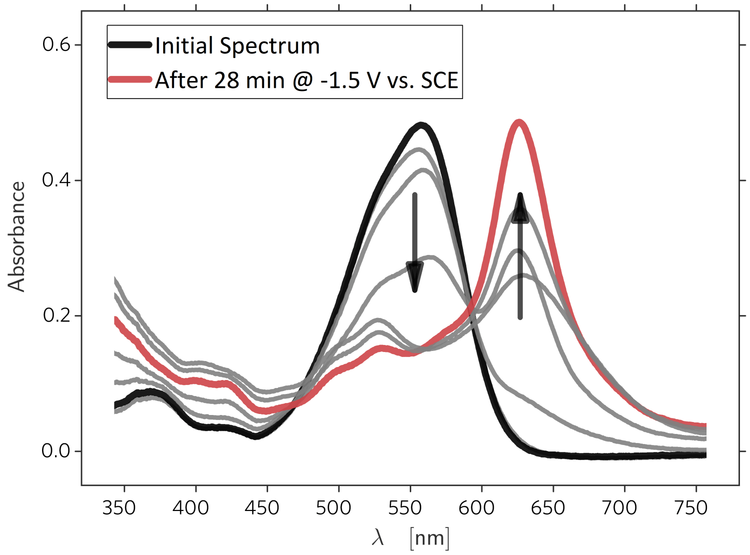

FIG. S5. Spectroelectrochemistry of PMI in DCM solution with Bu_4NPF_6 and SCE as reference electrode.

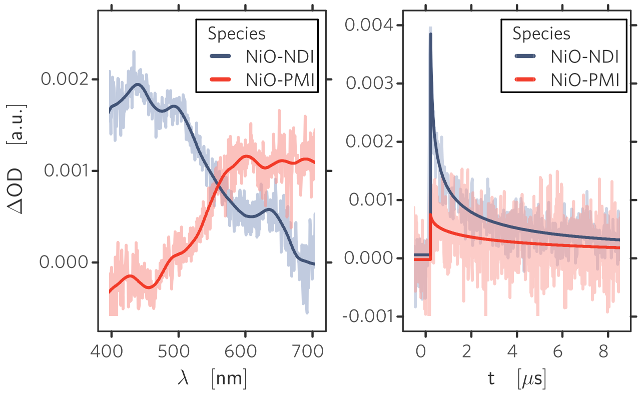

FIG. S6. Nanosecond transient absorption spectra and the ESA decay of dye anions of NiO-PMI (excited at 550 nm of 19.5 mJ) and NiO-NDI (excited at 450 nm of 20.1 mJ)

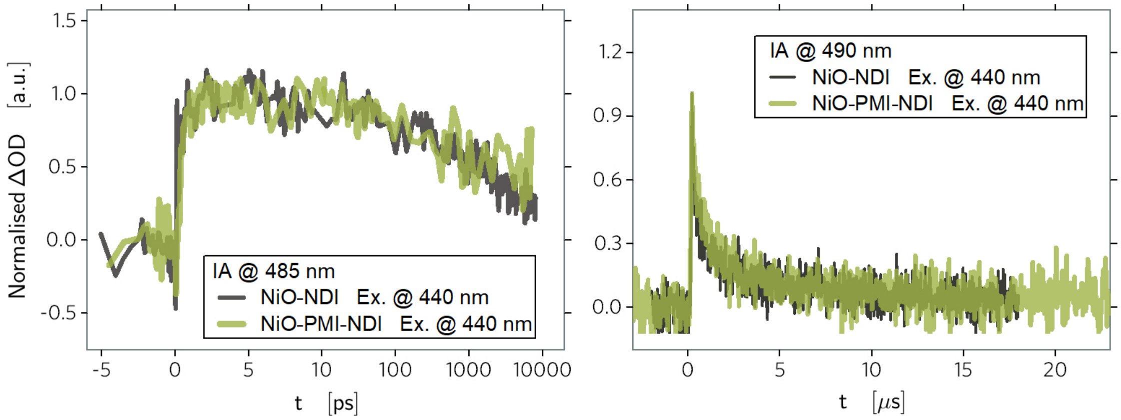

FIG. S7. Transient absorption decay at 490 nm (IA) of NiO-NDI and NiO-PMI-NDI excited at 440 nm.

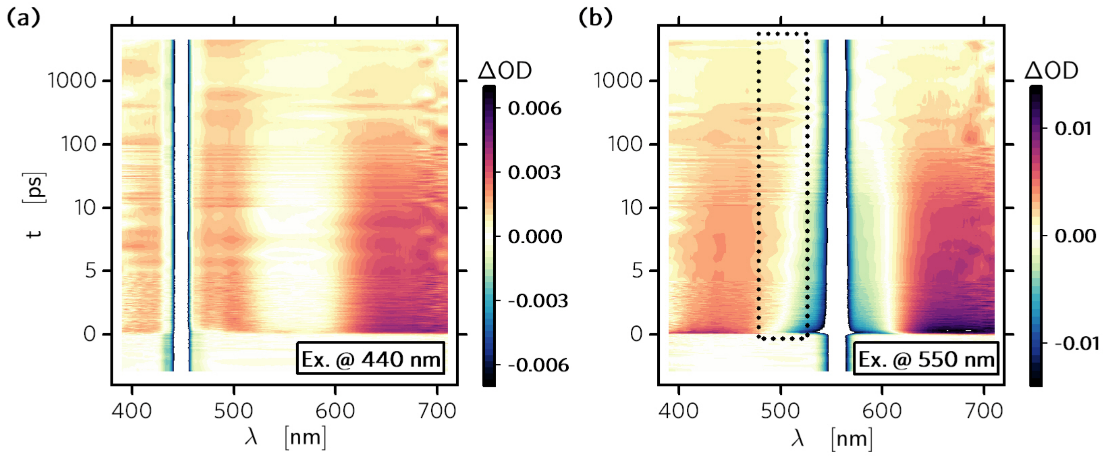

FIG. S8. Femtosecond transient absorption map of NiO-PMI-NDI film excited at 440 nm and 550 nm.

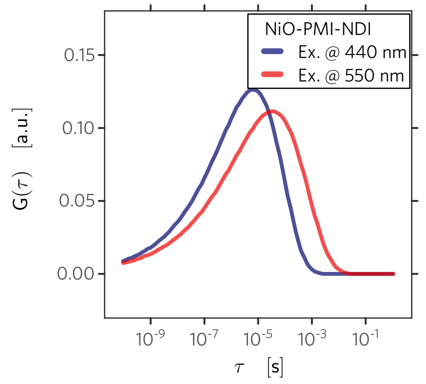

FIG. S9. Lévy distribution of the fitted lifetimes of NiO-PMI-NDI exicited at different chromophores (550 nm: $\tau = 16.5\ \mu$s, $\beta = 0.281$; 440 nm: $\tau = 3.19\ \mu$s, $\beta = 0.312$).

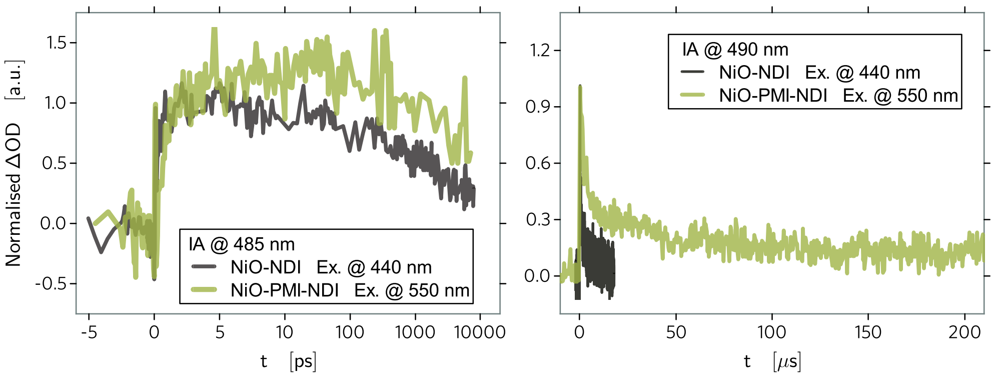

FIG. S10. Transient absorption decay at 490 nm of NiO-NDI excited at 440 nm and NiO-PMI-NDI excited at 550 nm.

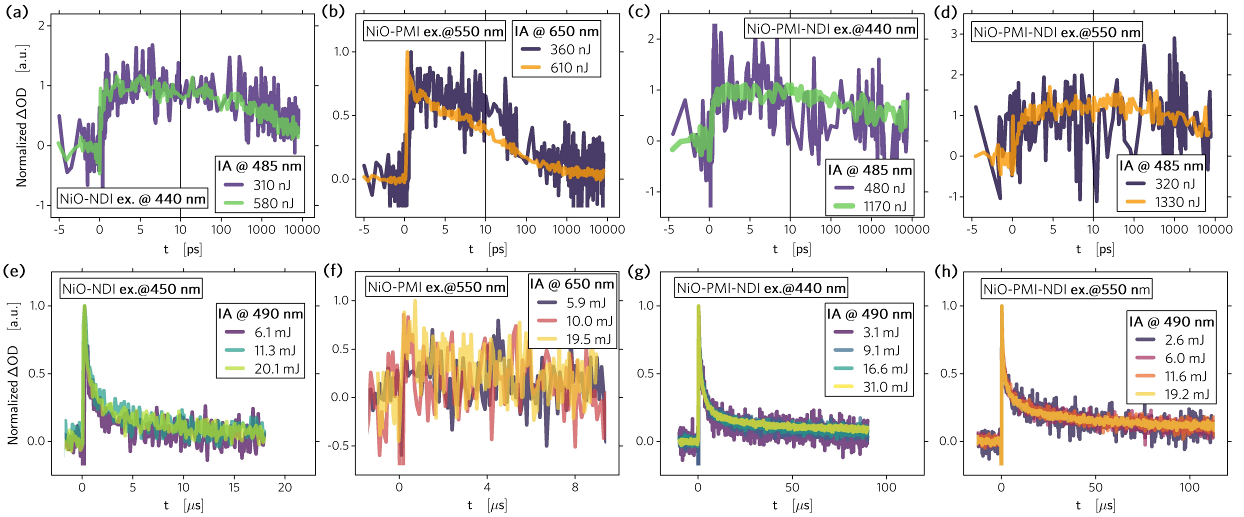

FIG. S11. Time resolved transient absorption decay of testes samples at different excitation intensities.

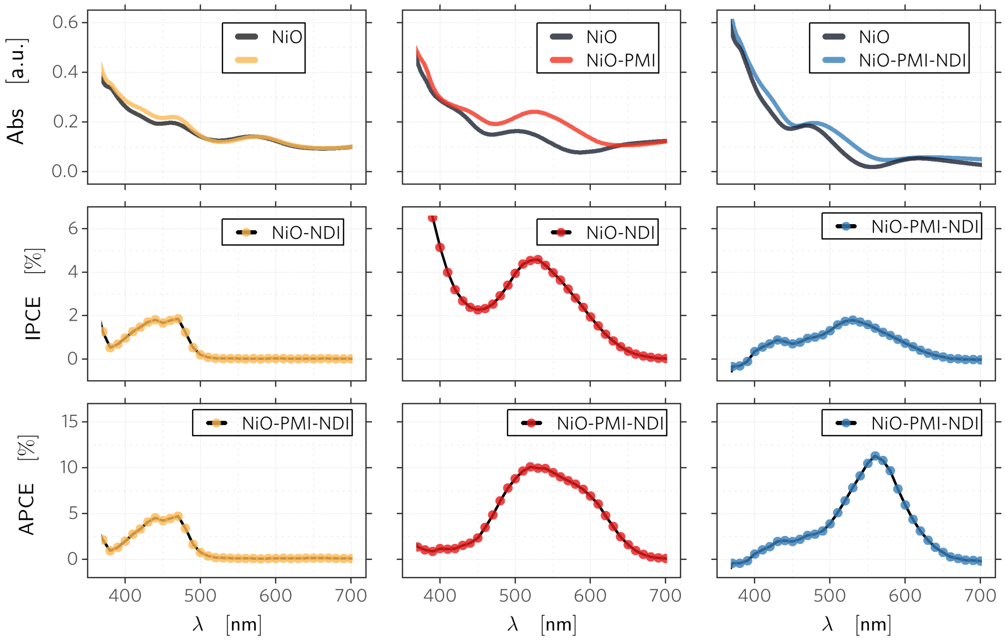

FIG. S12. Absorption spectra of NiO-NDI, NiO-PMI, and NiO-PMI-NDI film and the corresponding IPCE subtracting the NiO background. The electrolyte is composed of 1.0 M LiI and 0.1 M I_2 in acetonitrile.

TABLE S1. Fit parameters in the work

	Kinetic Pamaters						Index Information	
Decay Lines	A_1	τ_1	A_2	τ_2	A_3	τ_3	Method	Location
PMI-730	0.0012	3.0	0.0016	317	0.0050	3504	M.E.[a]	Fig. 2(e)
NiO-PMI-630	0.0023	97.2	0.0038	6.7	0.0007		M.E.	Fig. 2(f)
NDI-550	0.0042	1147	0.0037	17489	-	-	M.E.	Fig. 2(g)
NiO-NDI-550	0.0025	1.2	0.0011	42.4	0.0010	5771	M.E.	Fig. 2(h)
NiO-NDI-485	-0.0011	1.3	0.0009	335	0.0011	3863	M.E.	Fig. 2(h)
Ternary[b]-630	0.0027	1.0	0.0023	24.6	0.0018	771	M.E.	Fig. 3(c)
Ternary-485	-0.0027	23.7	0.0060	81.5	0.0017	8277	M.E.	Fig. 3(c)
Ternary-440ex	-0.0009	1.2	0.0007	51.7	0.0011	16889	M.E.	Fig. 3(d)
Ternary-550ex	-0.0027	23.7	0.0060	81.5	0.0017	8277	M.E.	Fig. 3(d)
PMI	0.168	31.1	0.7270	3134	-	-	M.E.	Fig. S5
PMI+NDI	0.336	1.0	0.571	4.6	0.260	2630	M.E.	Fig. S5
	τ		β		$\langle\tau\rangle$			
Ternary-440ex	3.2		0.312		24.8		KWW[c]	Fig. 3(e)
Ternary-550ex	16.5		0.281		208		KWW	Fig. 3(e)
NiO-NDI	2.5		0.293		12.1		KWW	Fig. S3
NiO-PMI	3.2		0.381		25.6		KWW	Fig. S3

[a] M.E. stands for Multiple Exponential

[b] Ternary stands for NiO-PMI-NDI

[c] KWW stands for Kohlrausch–Williams–Watts function.

[1] L. Tian, R. Tyburski, C. Wen, R. Sun, M. Abdellah, J. Huang, L. D'Amario, G. Boschloo, L. Hammarström, and H. Tian, Understanding the role of surface states on mesoporous nio films, J. Am. Chem. Soc. **142**, 18668 (2020).

[2] A. L. Smeigh, L. L. Pleux, J. Fortage, Y. Pellegrin, E. Blart, F. Odobel, and L. Hammarström, Ultrafast recombination for nio sensitized with a series of perylene imide sensitizers exhibiting marcus normal behaviour, Chem. Commun. **48**, 678 (2012).

[3] R. Haldar, A. Mazel, R. Joseph, M. Adams, I. A. Howard, B. S. Richards, M. Tsotsalas, E. Redel, S. Diring, F. Odobel, and C. Wöll, Excitonically coupled states in crystalline coordination networks, Chemistry – A European Journal **23**, 14316 (2017).

[4] G. Williams and D. C. Watts, Non-symmetrical dielectric relaxation behaviour arising from a simple empirical decay function, Trans. Faraday Soc. **66**, 80 (1970).

[5] C. P. Lindsey and G. D. Patterson, Detailed comparison of the williams–watts and cole–davidson functions, J. Chem. Phys. **73**, 3348 (1980).

[6] L. van Wilderen, C. Lincoln, and J. van Thor, Modelling multi-pulse population dynamics from ultrafast spectroscopy, PLoS ONE **6**, 10.1371/journal.pone.0017373 (2011).

[7] A. L. Smeigh, L. L. Pleux, J. Fortage, Y. Pellegrin, E. Blart, F. Odobel, and L. Hammarström, Ultrafast recombination for nio sensitized with a series of perylene imide sensitizers exhibiting marcus normal behaviour, Chem. Commun. **48**, 678 (2012).
